# Automatic flow analysis of digital subtraction angiography using independent component analysis in patients with carotid stenosis

**DOI:** 10.1371/journal.pone.0185330

**Published:** 2017-09-26

**Authors:** Han-Jui Lee, Jia-Sheng Hong, Chung-Jung Lin, Yi-Hsuan Kao, Feng-Chi Chang, Chao-Bao Luo, Wei-Fa Chu

**Affiliations:** 1 Department of Radiology, Taipei Veterans General Hospital, Taipei, Taiwan; 2 School of Medicine, National Yang-Ming University, Taipei, Taiwan; 3 Department of Biomedical Imaging and Radiological Sciences, National Yang-Ming University, Taipei, Taiwan; Universitatsklinikum Freiburg, GERMANY

## Abstract

**Purpose:**

Current time—density curve analysis of digital subtraction angiography (DSA) provides intravascular flow information but requires manual vasculature selection. We developed an angiographic marker that represents cerebral perfusion by using automatic independent component analysis.

**Materials and methods:**

We retrospectively analyzed the data of 44 patients with unilateral carotid stenosis higher than 70% according to North American Symptomatic Carotid Endarterectomy Trial criteria. For all patients, magnetic resonance perfusion (MRP) was performed one day before DSA. Fixed contrast injection protocols and DSA acquisition parameters were used before stenting. The cerebral circulation time (CCT) was defined as the difference in the time to peak between the parietal vein and cavernous internal carotid artery in a lateral angiogram. Both anterior-posterior and lateral DSA views were processed using independent component analysis, and the capillary angiogram was extracted automatically. The full width at half maximum of the time—density curve in the capillary phase in the anterior-posterior and lateral DSA views was defined as the angiographic mean transient time (aMTT; i.e., aMTT_AP_ and aMTT_Lat_). The correlations between the degree of stenosis, CCT, aMTT_AP_ and aMTT_Lat_, and MRP parameters were evaluated.

**Results:**

The degree of stenosis showed no correlation with CCT, aMTT_AP_, aMTT_Lat_, or any MRP parameter. CCT showed a strong correlation with aMTT_AP_ (r = 0.67) and aMTT_Lat_ (r = 0.72). Among the MRP parameters, CCT showed only a moderate correlation with MTT (r = 0.67) and Tmax (r = 0.40). aMTT_AP_ showed a moderate correlation with Tmax (r = 0.42) and a strong correlation with MTT (r = 0.77). aMTT_Lat_ also showed similar correlations with Tmax (r = 0.59) and MTT (r = 0.73).

**Conclusion:**

Apart from vascular anatomy, aMTT estimates brain parenchyma hemodynamics from DSA and is concordant with MRP. This process is completely automatic and provides immediate measurement of quantitative peritherapeutic brain parenchyma changes during stenting.

## Introduction

X-ray digital subtraction angiography (DSA), with its submillimeter and subsecond resolutions, is the gold standard for diagnosing cerebrovascular diseases [1–3]. Previous studies have demonstrated the feasibility of time—density curve (TDC) analysis, computed fluid dynamic simulation, and optic flow methods for quantitative hemodynamic measurement in DSA [4–7]. Computed fluid dynamic simulation estimates blood flow based on detailed anatomic and physiologic modeling and is therefore a time-consuming process. Nevertheless, its accuracy enables the successful prediction of aneurysmal rupture risk, and it is a promising method for risk stratification [6]. The optic flow method, which detects the movement of a contrast bolus between different frames to estimate velocity, is compute-intensive [5,8,9]. TDC analysis is the sole algorithm fulfilling the requirements of being reasonably fast, accurate, and automatic and is thus able to serve as an in-room hemodynamic assessment tool.

TDC analysis represents changes in the dynamic intensity of a contrast bolus passing through the region of interest (ROI) in DSA. It is affected by the bolus characteristics and pathologic conditions (e.g., arterial stenosis or arterio-venous shunts) [10,11]. Regarding its advantages, TDC analysis is less computer intensive, measurement outcomes are immediate [5,8,9], and no additional radiation is required [10,12,13].

TDC analysis makes it possible to measure time to peak (TTP; i.e., the time point at which the ROI achieves the highest concentration). Cerebral circulation time (CCT) is defined as the difference between the TTPs of the internal carotid artery and the parietal vein (PV). The PV is a relatively stable cortical vein located in the vicinity of the superior sagittal sinus, compared with the major venous sinuses. Therefore, it was chosen as the reference for measuring the time required for blood to pass through the brain [14]. The CCT has been shown to be an effective method for quantifying the intravascular flow in different vascular disorders such as carotid stenosis, carotid cavernous fistula, and vasospasm peritherapeutically [13–15].

One drawback of CCT is that it requires manual selection of the internal carotid artery and PV, rendering the measurement process susceptible to intraobserver and interobserver variations and potentially delaying the workflow of endovascular treatment [12]. To establish a more objective measurement, in a previous study, we introduced independent component analysis (ICA) to avoid the manual selection of ROIs. ICA decomposes the mixed signals into statistically independent components [16]. The superiority of ICA for estimating individual components with partial volume effects in magnetic resonance perfusion (MRP) images has been demonstrated [17,18]. Furthermore, a deconvolution approach can estimate cerebral blood flow, cerebral blood volume, and mean transit time from DSA imaging [19]. Through the automatic generation of an angiographic surrogate to assemble the CCT, the operator can save the time required for post processing and focus on the operation. Thus, the aim of the current study was to develop a real-time automatic computerized algorithm to optimize an angiographic imaging marker for generating a surrogate for brain parenchyma perfusion. This algorithm was compared with manually defined CCT and MRP.

## Material and methods

### Patient selection

The Taipei Veterans General Hospital institutional review board approved this retrospective study. The requriement for patient consent was waived (TVGH IRB No. 2014-16-014) because all the data were anonymized. From January 2012 to September 2016, 136 patients with extracranial internal carotid stenosis (>70%, North American Symptomatic Carotid Endarterectomy Trial [NASCET]) were referred to our department for carotid stenting and were included in this study. Patients with contralateral or distal stenosis (n = 15), those with previous large territorial infarcts (n = 2), those without preoperative MRP imaging (n = 63), and those without complete intracranial angiographic series (n = 12) were excluded. The remaining 44 patients were included in this study.

### DSA imaging protocol

For all 44 patients, DSA acquisitions were performed using a standard, clinically routine protocol on the same biplane AngioSuite (AXIOMArtis^®^, Siemens Healthcare, Forchheim, Germany). The degree of arterial stenosis was determined by the more severe degree of the anterior-posterior (AP) or lateral views according to NASCET criteria [20]. A power injector (Liebel-FlarsheimAngiomat^®^, Illumena, San Diego, USA) was used to administer a contrast bolus by placing a 4-F angiocatheter in the common carotid artery at the C4 vertebral body level. A bolus of 12 mL of 60% diluted contrast medium (340 mg I/mL) was then administered within 1.5 seconds. No additional contrast medium or radiation was used. The acquisition parameters were 7.5 frames per second for the first 5 seconds, followed by 4 frames per second for 3 seconds, 3 frames per second for 2 seconds, and finally 2 frames per second for 2 seconds. The entire DSA acquisition lasted 12 seconds, but was manually prolonged to visualize internal jugular vein opacification in cases of slow intracranial circulation [12].

### Cerebral circulation time

All angiographic datasets were sent to a workstation equipped with commercialized software (Syngo iflow^®^, Siemens Healthcare, Forchheim, Germany). A TDC was generated according to ROIs manually selected in the angiographic series. The TTP was defined as the time point at which individual ROIs achieved the maximum concentration. Moreover, the CCT, defined as the difference in TTP between the PV and the cavernous segment of the internal carotid artery, was manually determined by an interventionist with 12 years of experience. CCT has been demonstrated to have a strong correlation with the mean transit time of MRP, and it reflects hemodynamic changes during treatment of various cerebrovascular diseases in the angio-room [15].

### Angiographic mean transit time

**Fig 1:**
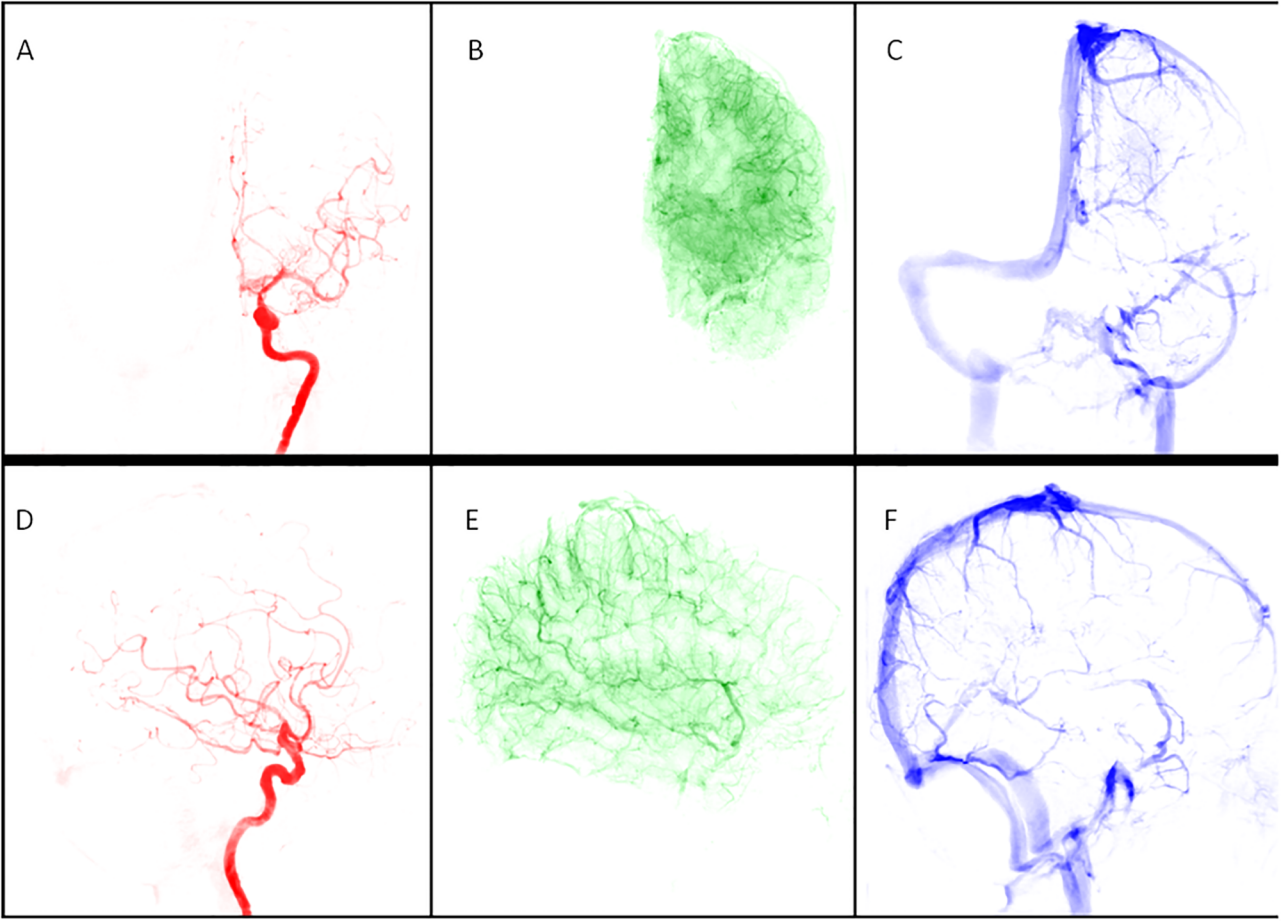
A 67-year-old male patient with 84% left carotid stenosis received carotid angiogram before stenting. The angiogram was processed using independent component analysis with motion correction and automatically produced (A) arterial, (B) capillary, and (C) venous phases of the anterior-posterior view and (D) arterial, (E) capillary, and (F) venous phases of the lateral view.

**Fig 2:**
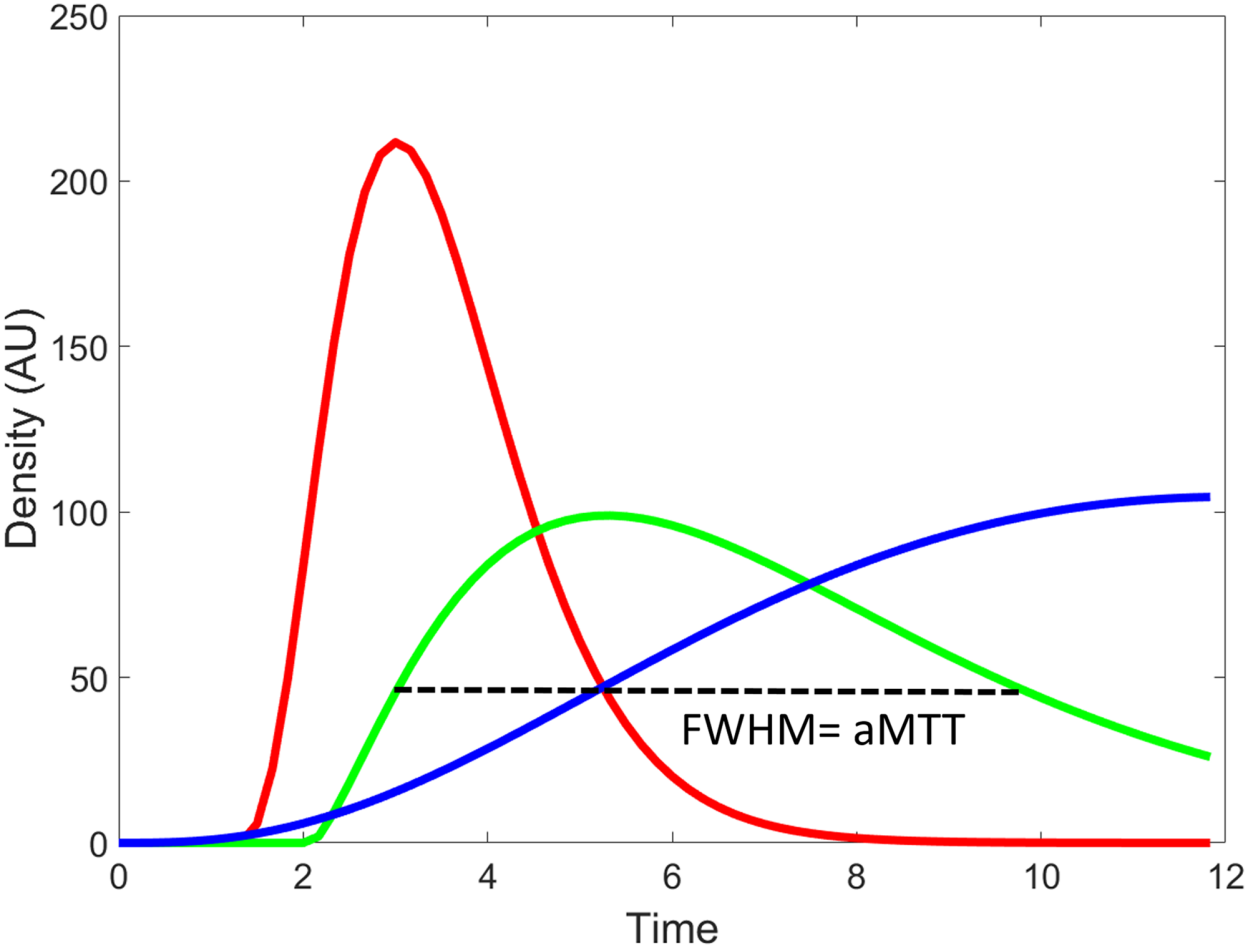
Time—Density curves of arterial (red), capillary (green), and venous (blue) phases of a lateral view of the lesional carotid angiogram of a 67-year-old male patient with 83% carotid stenosis. The full width at half maximum (FWHM) of the time—density curve in the capillary phase was defined as the angiographic mean transient time (aMTT).

The dataset was post processed using a Pentium-based personal computer. All DSA analyses were performed using in-house software written in MATLAB (MathWorks, Natick, MA). We applied the scale-invariant feature transform technique by aligning all other images of a series to the reference image (at a time of 0) to reduce motion artifacts in sequential dynamic subtraction X-ray projection images [21]. ICA is a data-driven method that entails decomposing mixed signals into statistically independent components, and it can efficiently and objectively reflect cerebral hemodynamic changes [22,23]. In this experiment, we permuted the 2D images of DSA into a 1D signal, and used the FastICA technique [16] on a patient basis. The number of output IC components was set to three: arterial, capillary, and venous components (Fig 1). Thresholds were determined using Ostu’s intervariance maximization technique to generate the vessel masks [24]. A TDC in the capillary phase was extracted by multiplying the vessel mask to the DSA series on a pixel-by-pixel basis, and the curve was fitted by the gamma-variate function. Angiographic mean transient time was used to define the full width at half maximum of the TDC derived from the capillary component in the AP and lateral views, labelled aMTT_AP_ and aMTT_Lat_, respectively (Fig 2).

### Magnetic resonance perfusion

All MRP processes were performed one day before carotid stenting on the same 1.5 Tesla scanner (Signa HDxt^®^, GE Healthcare, Milwaukee, USA) with an eight-channel neurovascular coil. Imaging parameters were as follows: 60° flip angle, 1000-millisecond TR/40-millisecond TE, 7-mm section thickness with a 7-mm imaging gap, 240-mm field of view, and 128 × 128 acquisition matrix. The MR scan and bolus administration were started simultaneously. Perfusion Mismatch Analyzer software (version 5.0, ASIST Group, Japan) was used for analysis [25]. The arterial input function of the ipislateral middle cerebral artery was chosen. Standard singular value decomposition was used as the deconvolution algorithm for obtaining Tmax, Cerebral blood volume (CBV), Cerebral blood flow (CBF), and Mean transient time (MTT) values. The ROIs in MRP were designated to include ipsilateral middle cerebral artery cortical territories (i.e., M1, M2, and M3) according to the Alberta Stroke Program Early CT Score criteria [26–28].

### Statistical analyses

All statistical analyses were performed using SPSS 20 (2010; IBM-SPSS, Chicago, IL). Pearson correlations were calculated between the degree of stenosis (%), CCT, aMTT, and MRP parameters and all indices for the three phases (artery, capillary and venous). Significance was set at *p* < 0.05.

## Results

**Fig 3:**
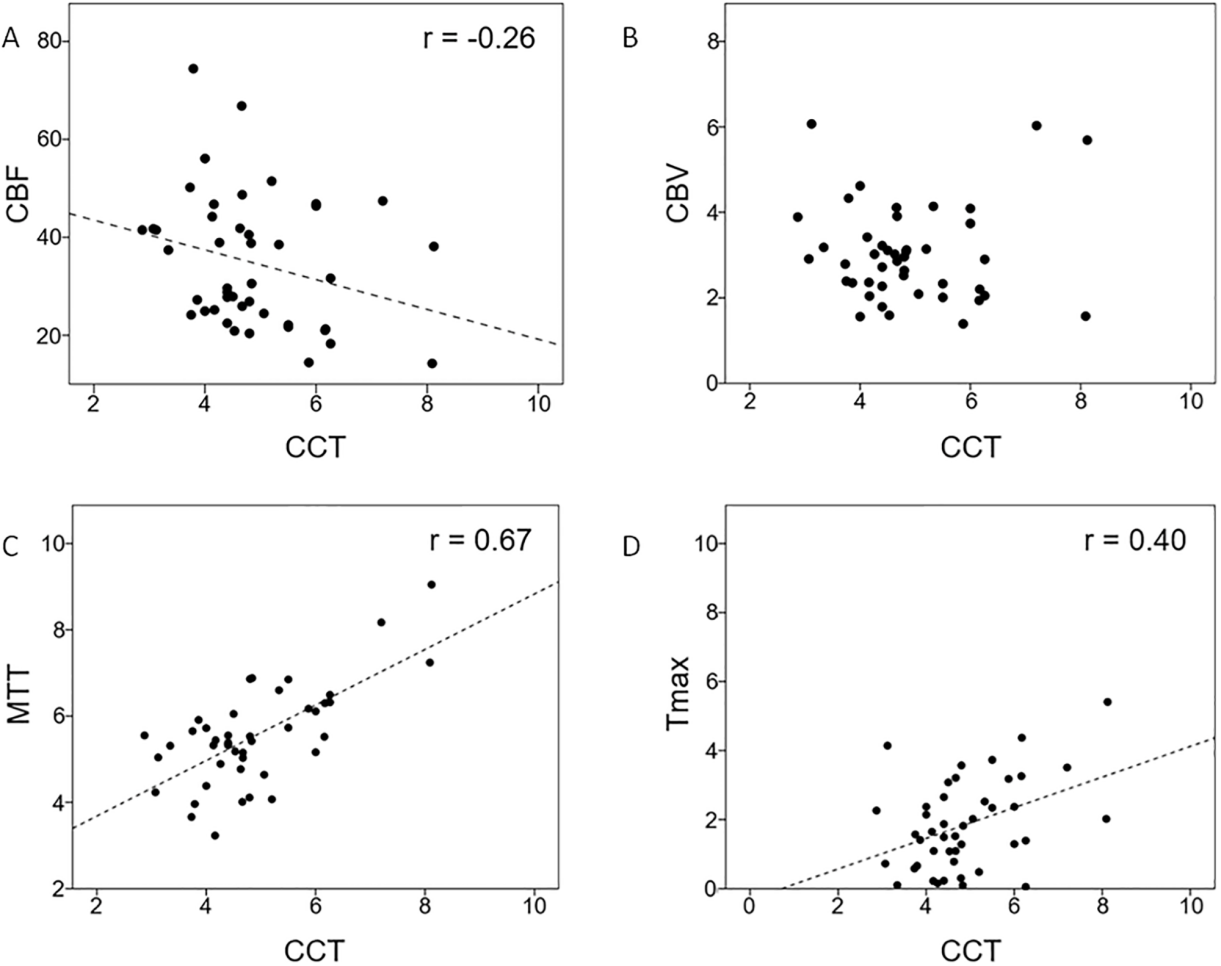
Correlation between cerebral circulation time (CCT) and (A) CBF, (B) CBV, (C) MTT, and (D) Tmax.

**Fig 4:**
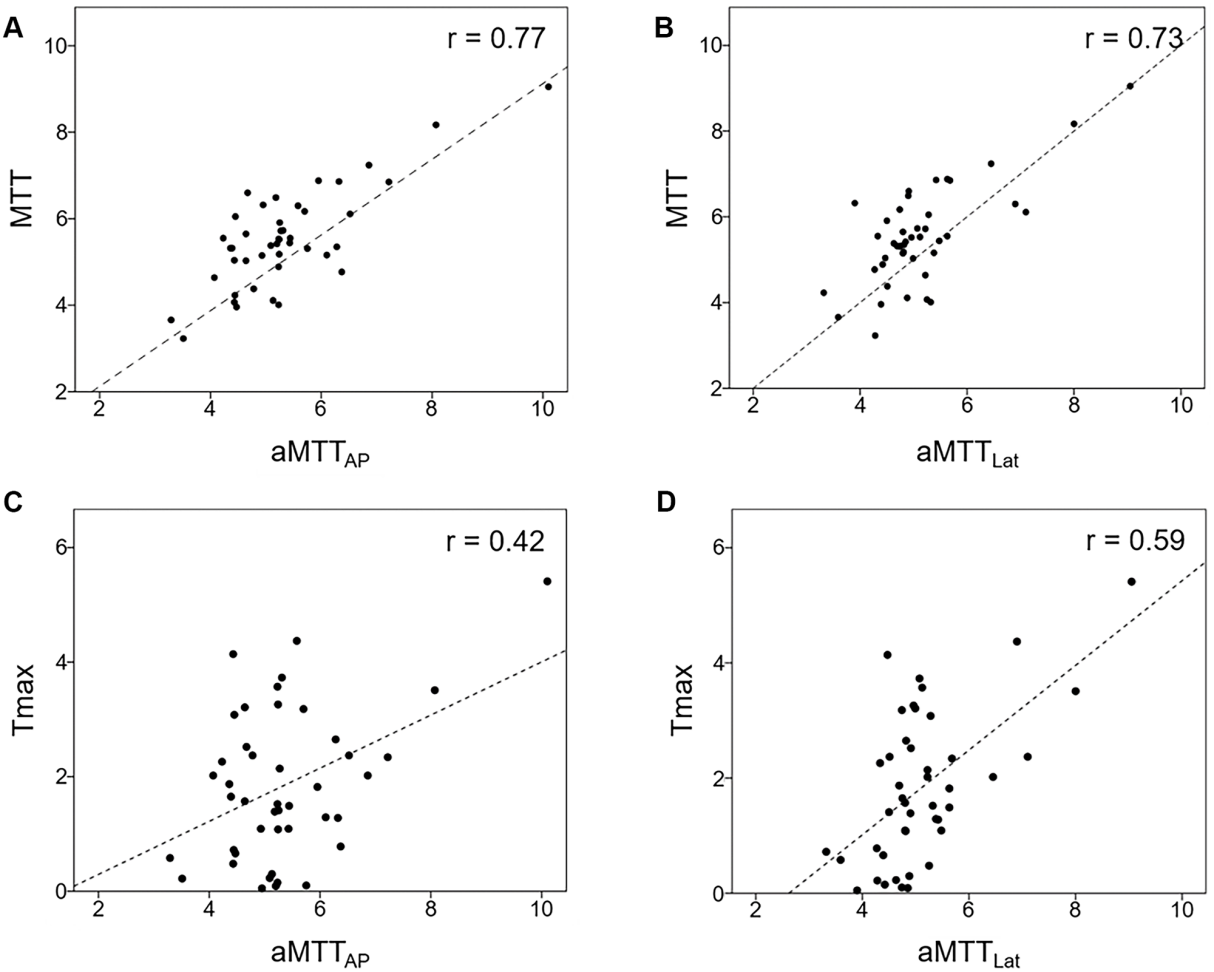
Correlation between (A) MTT and aMTT_AP_; (B) MTT and aMTT_Lat_; (C) Tmax and aMTT_AP_; and (D) Tmax and aMTT_Lat_.

Patient characteristics, angiographic parameters, and results of MR perfusion are listed in Table 1. None of the patients experienced acute strokes peritherapeutically, as revealed by MR. The degree of stenosis showed no correlation with the CCT, aMTT_AP_, aMTT_Lat_, or any MRP parameter. The average aMTT_AP_ and aMTT_Lat_ values for the whole study group were 5.34 ± 1.18 seconds and 5.12 ± 1.04 seconds respectively. The average CCT was 4.87 ± 1.19 seconds. CCT showed a strong correlation with aMTT_AP_ (r = 0.67) and aMTT_Lat_ (r = 0.72). Among the MRP parameters, the CCT only showed a moderate correlation with time-dependent parameters, namely MTT (r = 0.67) and Tmax (r = 0.40) (Fig 3). Moreover, aMTT_AP_ showed a moderate correlation with Tmax (r = 0.42) and a strong correlation with MTT (r = 0.77); aMTT_AP_ was not correlated with CBF or CBV. Finally, aMTT_Lat_ showed a moderate correlation with Tmax (r = 0.59) and a strong correlation with MTT (r = 0.73; Fig 4).

**Table 1:** Patient characteristics, angiographic parameters, and results of MR perfusion of 44 patients receiving carotid stenting.

Patient with unilateral carotid stenosis	
**Number**	44
**Age**	72.4 ± 11.8
**Heart rate (beat/minutes)**	65.3 ± 19.4
**Blood pressure (mmHg)**	93.2 ± 2l.2
**Stenotic degree (%)**	79.3 ± 8.3%
**Prior lacunar infarct**	8 (16%)
**aMTT**_**AP (Second)**_	*5*.*3*4 ± *1*.18
**aMTT**_**Lat**_ **(Second)**	*5*.1*2* ± *1*.04
**CCT (Second)**	4.87 ± *1*.19
**CBF (%)**	34.78 ± 13.54
**CBV (ml/100g)**	3.03 ± *1*.13
**MTT (Second)**	5.53 ± *1*.14
**Tmax (Second)**	1.84 ± *1*.31

## Discussion

Cerebral perfusion has been reported to be influenced by multiple factors such as proximal luminal stenosis, collateral circulation, and brain parenchyma viability [29,30]. Therefore, the carotid degree of stenosis alone could not approximate cerebral perfusion deficits. No correlation was observed between any MRP parameter and the degree of stenosis in the current study, confirming that stenosis is a poor indicator of ischemia. Moreover, we revealed that automatic outputs from customized software (aMTT_AP_ and aMTT_Lat_) and the CCT showed a strong correlation with MTT (r = 0.67–0.72). According to Soinne et al., MTT is the most sensitive MRP parameter for the detection of reduced cerebral perfusion in carotid stenosis [31]. Both aMTT parameters (aMTT_AP_ and aMTT_Lat_) measured the duration for which contrast-containing blood flow remained in the capillary, the smallest vasculature identifiable and conceptually closest to brain parenchyma in DSA imaging. Therefore, aMTT_AP_ and aMTT_Lat_ in DSA resemble MTT in MRP.

Tmax is a physiologic measurement comprising intravascular components (poststenotic delay and dispersion effect) and a parenchyma component (mean transient time) [25,32,33]. The complex physiologic nature of Tmax explains why its correlation with aMTT_AP_ and aMTT_Lat_ was not as strong as that with MTT. Nevertheless, quantifying CBF and CBV in the two-dimensional domain of DSA is challenging because of the difficulty associated with volumetric measurements and the overlying vasculature [27]. Through this automatic in-room TDC assessment, we could use Tmax, adjunct to MTT, as an angiographic marker for detecting perfusion deficits and evaluating the peritherapeutic treatment effect [34].

**Fig 5:**
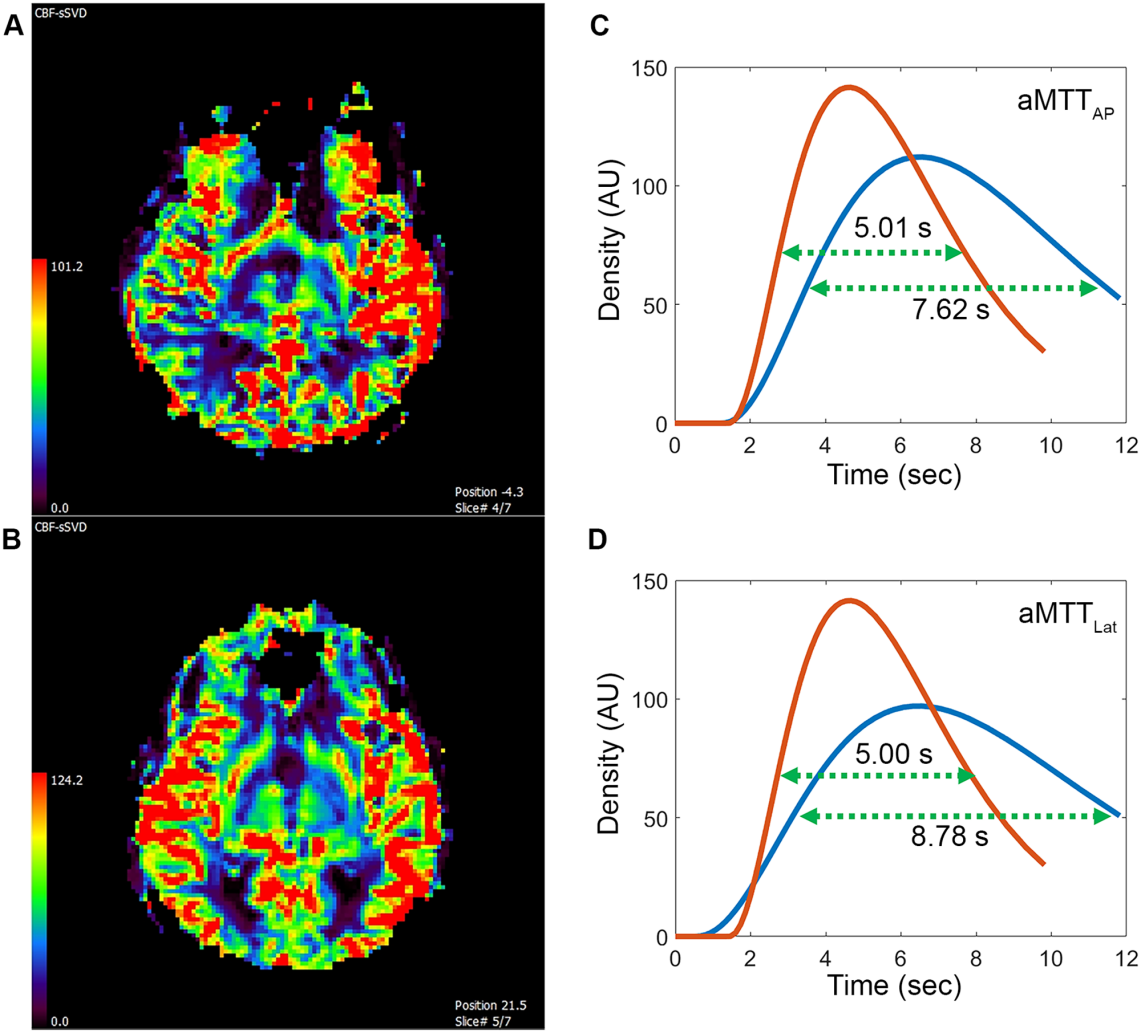
A 92-year-old female with 80% stenosis of left internal carotid artery received carotid stenting. (A) Pre-stenting MR perfusion showed decreased cerebral blood flow in the right hemisphere compared to the left hemisphere. Twelve hours after the procedure, the patient experienced headache and seizure. (B) Emergent MR perfusion after stenting showed 56% increased cerebral blood flow in the right hemisphere compared to the left hemisphere. Based on clinical features and MR perfusion, this patient developed hyperperfusion syndrome. (C) Post-stenting aMTT_AP_ (red) was shorter than pre-stenting aMTT_AP_ (blue) by 2.61 seconds, indicative of excessive increased blood flow. (D) Post-stenting aMTT_Lat_ (red) showed the same trend of increased blood flow and was shorter than pre-stenting aMTT_Lat_ (blue) by 3.78 seconds. Blood pressure was strictly controlled under 120 mm Hg and the symptoms gradually subsided in the following days.

A shortened CCT during carotid stenting is a sign of CBF restoration in patients receiving carotid stenting [12,35]. Furthermore, hyperperfusion syndrome has been reported to be the most catastrophic adverse event associated with carotid stenting and to be associated with a prolonged CCT [36–39]. Given the high correlation between both aMTT parameters and the CCT, we expect that spontaneously generated angiographic parameters can be used to identify patients at a higher risk of hyperperfusion syndrome, thus avoiding complications in the angio-room (Fig 5). Moreover, the characteristic spontaneously generated parameters from our customized program can facilitate the workflow of endovascular treatments. Flat-panel perfusion imaging alternatively provides estimation of cerebral hemodynamics, but the risk of additional iodine and radiation exposure should be carefully weighed [40,41].

Although infrequent, more arterioles have been observed in the capillary phase in cases of severe stenosis (more than 90%) than in cases of moderate stenosis (70%–90%). Martel et al. similarly noticed a tendency towards a more heterogenous dataset in more stenotic patients in MRP [42]. We hypothesized that the stagnant arterial flow in cases of severe carotid stenosis mimics the flow pattern of the capillary flow and thus generates a mixture of arteries and capillaries. Different threshold methods might improve the correct segmentation of stagnant flow. This question warrants further study.

This study has several limitations. First, the overlapping of anatomical structures complicated the TDCs in the two-dimensional domain of DSA, thus resulting in some inaccuracies in volumetric estimation. Second, the accuracy of segmentation of the three phases decreased as the motion artifacts increased. This phenomenon occurs because the artifacts generated a sharp density change in the TDC, thus simulating a distinguished curve, which interfered with subsequent segmentation processes. Optimizing motion correction algorithms is warranted in further applications. Third, our methodology is based on TDC analysis and is therefore subjective in terms of detecting territorial flow changes. Subtle changes of region flow fields are beyond the scope of this methodology. Fourth, generalization of this approach should be carefully executed because varying injection rates, durations, and DSA acquisition frame rates might potentially influence the value of angiographic mean transit times. However, as long as the region of interest is ten times of the vessel diameter downstream to the catheter tip, the contrast has been shown to mix thoroughly with the blood flow under clinical injection protocols and is thus able to represent physiologic flow hemodynamically [43].

## Conclusion

Our study confirmed that ICA provides an efficient and completely automatic analysis of DSA. Both aMTT parameters were highly correlated with MTT and Tmax in MRP. This study showed that these parameters can provide objective, immediate cerebral hemodynamic measurements and effectively reflect the severity of perfusion deficits in carotid stenosis. TDC analysis can serve as an in-room peritherapeutic hemodynamic monitoring tool and does not require additional radiation or contrast, and it potentially facilitates one-stop shop imaging in thrombectomy for acute infarcts.

## Supporting information

S1 File Original dataset. (XLSX)
